# Central ocular motor disorders, including gaze palsy and nystagmus

**DOI:** 10.1007/s00415-014-7385-9

**Published:** 2014-08-22

**Authors:** M. Strupp, O. Kremmyda, C. Adamczyk, N. Böttcher, C. Muth, C. W. Yip, T. Bremova

**Affiliations:** 1 Department of Neurology and German Center for Vertigo and Balance Disorders, University Hospital Munich, Campus Großhadern, Marchioninistrasse 15, 81377 Munich, Germany; 2 National Neuroscience Institute and Department of Neurology, Singapore General Hospital, Singapore, Singapore

**Keywords:** Ocular motor, Examination, Neurodegenerative disorder, Diagnosis, Treatment

## Abstract

An impairment of eye movements, or nystagmus, is seen in many diseases of the central nervous system, in particular those affecting the brainstem and cerebellum, as well as in those of the vestibular system. The key to diagnosis is a systematic clinical examination of the different types of eye movements, including: eye position, range of eye movements, smooth pursuit, saccades, gaze-holding function and optokinetic nystagmus, as well as testing for the different types of nystagmus (e.g., central fixation nystagmus or peripheral vestibular nystagmus). Depending on the time course of the signs and symptoms, eye movements often indicate a specific underlying cause (e.g., stroke or neurodegenerative or metabolic disorders). A detailed knowledge of the anatomy and physiology of eye movements enables the physician to localize the disturbance to a specific area in the brainstem (midbrain, pons or medulla) or cerebellum (in particular the flocculus). For example, isolated dysfunction of vertical eye movements is due to a midbrain lesion affecting the rostral interstitial nucleus of the medial longitudinal fascicle, with impaired vertical saccades only, the interstitial nucleus of Cajal or the posterior commissure; common causes with an acute onset are an infarction or bleeding in the upper midbrain or in patients with chronic progressive supranuclear palsy (PSP) and Niemann–Pick type C (NP-C). Isolated dysfunction of horizontal saccades is due to a pontine lesion affecting the paramedian pontine reticular formation due, for instance, to brainstem bleeding, glioma or Gaucher disease type 3; an impairment of horizontal and vertical saccades is found in later stages of PSP, NP-C and Gaucher disease type 3. Gaze-evoked nystagmus (GEN) in all directions indicates a cerebellar dysfunction and can have multiple causes such as drugs, in particular antiepileptics, chronic alcohol abuse, neurodegenerative cerebellar disorders or cerebellar ataxias; purely vertical GEN is due to a midbrain lesion, while purely horizontal GEN is due to a pontomedullary lesion. The pathognomonic clinical sign of internuclear ophthalmoplegia is an impaired adduction while testing horizontal saccades on the side of the lesion in the ipsilateral medial longitudinal fascicule. The most common pathological types of central nystagmus are downbeat nystagmus (DBN) and upbeat nystagmus (UBN). DBN is generally due to cerebellar dysfunction affecting the flocculus bilaterally (e.g., due to a neurodegenerative disease). Treatment options exist for a few disorders: miglustat for NP-C and aminopyridines for DBN and UBN. It is therefore particularly important to identify treatable cases with these conditions.

## Introduction

Many patients present with symptoms of blurred vision, reduced visual acuity, ‘bouncing images’ (oscillopsia) or double vision. Other patients complain of dizziness, vertigo, postural imbalance, tendency to fall or recurrent falls, gait disturbances or ataxia. These symptoms may indicate a dysfunction of the ocular motor system [1]. Unfortunately, they are often overlooked simply because patients are not adequately clinically examined, although an impairment of eye movements is important for the differential diagnosis and, in particular, to ascertain whether the brainstem or the cerebellum is affected. Further, the examination of eye movements is of clinical relevance for several disciplines, especially neurology, ophthalmology, pediatrics and neuropediatrics, internal medicine and otorhinolaryngology. In patients with an acute onset of the above-mentioned symptoms, the most important differential diagnosis is ischemia, bleeding or inflammation of the brainstem; they may also occur in Wernicke encephalopathy. If the symptoms are chronic or chronically progressive, possible causes can include metabolic, neurodegenerative, inherited or inflammatory disorders (multiple sclerosis or encephalitis) or tumors.

To classify the symptoms topographically, anatomically, a systematic clinical bedside examination of the different types of eye movements is mandatory, particularly to distinguish between central and peripheral ocular motor disorders [1, 2] and central and peripheral vestibular disorders [3, 4]. In this context, it is important to note that the bedside examination of eye movements, even without equipment-based additional investigations, is evidently even more sensitive for the diagnosis of acute vestibular syndromes and for differentiating between peripheral and central lesions than magnetic resonance imaging (including diffusion-weighted sequences) [5].

The diagnosis of an acute central disorder requires rapid admission to hospital as this may be caused by brainstem ischemia or bleeding. Clinical experience shows that the examination of patients with ocular motor disturbances presents a particular challenge for many clinicians for three reasons: first, the anatomy and physiology of the ocular motor, vestibular, and cerebellar systems are complex; second, the neurological and neuro-ophthalmological examinations require a systematic approach and an experienced diagnostic perspective; third, the interpretation requires an evaluation of all neuro-otological and neuro-ophthalmological findings within the context of the patient’s history. In addition to a precise topographic anatomical diagnosis of these disorders, one should focus on those forms of central ocular motor disorders and nystagmus that are treatable, such as downbeat nystagmus (DBN), upbeat nystagmus (UBN), Wernicke encephalopathy, Niemann–Pick disease type C (NP-C) and Gaucher disease type 3.

In the first part of this article, the different types of eye movements (along with their topographical-anatomical relevance), how to take a patient history and appropriate examination procedures are presented. The second part deals with the most common forms of central eye movement disorders and nystagmus. Any repetition is intentional as different perspectives based on clinical symptoms and functional anatomy are covered.

## Physiological forms of eye movements

Before we focus on pathological eye movements, we list here the physiological forms: (1) smooth pursuit, where the eye follows a moving target; (2) saccades, where the gaze rapidly jumps from one fixation point to another; (3) vergence eye movements (i.e. movements during which the eyes do not move in parallel but relative to one another); (4) vestibulo–ocular reflex (VOR); the signal triggering eye movements comes from the labyrinth, which keeps the image of the visual surroundings stable on the retina during head movements; (5) optokinetic reflex, which is triggered by moving visual targets and consists of smooth pursuit and saccades; and (6) gaze holding (i.e. the ability to keep the eyes in an eccentric position (see [1, 6]). With the exception of voluntary saccades and vergence/divergence saccades, all the other types of eye movements are reflexive movements. All these different types of eye movements serve to keep the visual target on the macula stable and thus avoid illusory movements (oscillopsia) and blurred vision under different conditions such as fixating a central or peripheral visual target, following a slowly or very quickly moving target, moving the head, or walking and running around.

## Patient history

Depending on the underlying cause, patients with ocular motor disturbances usually report the following symptoms in isolation or in combination: blurred vision, reduced visual acuity, double vision, jumping images (so-called oscillopsia) either while looking straight ahead, right/left or up/down (indicating an underlying nystagmus), or while turning the head or walking (indicating a deficit of the VOR), rotatory vertigo, postural imbalance, tendency to fall or brainstem-related symptoms (e.g., swallowing or speaking difficulties), cerebellar symptoms (e.g., coordination problems of the extremities), or symptoms of the inner ear (e.g., hearing loss or tinnitus).

## Clinical bedside examination of the ocular motor and vestibular system

A combined examination of these systems in patients with the above-mentioned symptoms is always necessary to make a correct anatomical diagnosis. An overview is given in Table 1.

**Table 1:** Overview of the examination of the ocular motor and the vestibular systems (modified from [3])

Type of examination	Question
Inspection	
Head/body posture	Tilt or turn of head/body
Position of eyelids	Ptosis
Eye position/motility	
Position of eyes during straight-ahead gaze	Misalignment in primary position, spontaneous or fixation nystagmus
	Horizontal or vertical misalignment
Cover/uncover test	
Examination of eyes in eight positions (binocular and monocular)	Determination of range of motility, gaze-evoked nystagmus (GEN), end-position nystagmus
Gaze-holding function	
10–40° in the horizontal or 10–20° in the vertical and back to 0°	GEN: horizontal and vertical, rebound nystagmus
Slow smooth pursuit movements	
Horizontal and vertical	Smooth or saccadic
Saccades	
Horizontal and vertical when looking around or at targets	Latency, velocity, accuracy, conjugacy
Optokinetic nystagmus (OKN)	
Horizontal and vertical with OKN drum or tape	Inducible, direction, phase (reversal or monocularly diagonal)
Peripheral vestibular function	
Head-impulse test for clinical examination of the VOR (Halmagyi–Curthoys test): rapid turning of the head and fixation of a stationary target	Unilateral or bilateral peripheral vestibular deficit
Fixation suppression of the VOR	
Turning the head and fixation of a target moving at same speed	Impairment of fixation suppression of the VOR
Examination with Frenzel’s glasses	
Straight-ahead gaze, to the right, to the left, downward and upward	Peripheral vestibular spontaneous nystagmus versus central fixation nystagmus
Head-shaking test	Head-shaking nystagmus

*GEN* gaze-evoked nystagmus, *OKN* optokinetic nystagmus, *VOR* vestibulo–-ocular reflex

### Head tilt

An abnormal position of the head toward the right or left shoulder is observed particularly in patients with paresis of the oblique eye muscles (e.g., palsy of the trochlear nerve or the superior oblique muscle, in which the head is turned to the non-affected side to lessen diplopia), or in those with an ocular tilt reaction (OTR) due to a tonus imbalance of the VOR in the roll plane [7, 8]. In the OTR the head is tilted to the side of the lower eye. A tilting of the head to the side of the lesion indicates either an acute unilateral peripheral vestibular lesion or an acute unilateral central lesion in the medulla oblongata (e.g., in Wallenberg’s syndrome) [9]. A head tilt to the contralateral side occurs in pontomesencephalic lesions [10, 11].

### Examination of the eye position during straight-ahead gaze with the cover tests

When examining the patient, attention should be paid to the primary position of the eyes when the patient looks straight ahead, when one eye is covered or when each eye is covered alternately (alternating cover test), that is parallel position or horizontal or vertical misalignment. These tests allow diagnosis of latent or manifest strabismus. The prerequisite for all cover tests is the presence of foveal fixation.

### The one-eye cover test

**Fig. 1:**
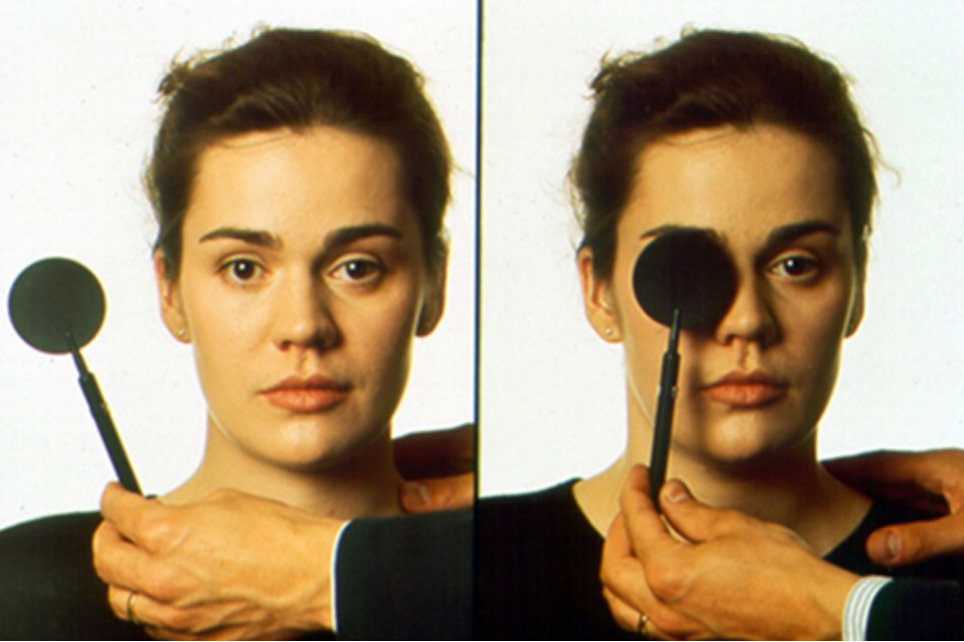
Cover and uncover test examination: examination to detect misalignments of the visual axes (modified from [3])

In the one-eye cover test heterotropia (i.e. manifest strabismus) can be observed in the uncovered eye; the latter moves when the other eye is covered (Fig. 1). Heterotropia is defined as a misalignment of the visual axes, even during binocular fixation. First, the patient is asked to fixate either a near target (at a distance of 30–40 cm) or one 5–6 m away. Then the examiner covers one eye and looks for correction movements of the now uncovered eye. If the uncovered eye moves: (a) from the inside outward, esotropia is present; (b) from the outside inward, exotropia; (c) from above downward, hypertropia and; (d) from below upward, hypotropia. The other eye is then examined. An acute vertical divergence (so-called skew deviation; one eye is higher than the other) indicates a central lesion of graviceptive pathways as part of the so-called OTR.

### The one-eye cover/uncover test

The one-eye cover/uncover test is used to prove the presence of heterophoria (i.e. latent strabismus) (Fig. 1). This is a misalignment of the eye axes when a target is fixated with one eye only. It is important to perform the above-mentioned cover part of the test before the cover/uncover part to first exclude heterotropia. First, one eye is covered for about 10 s, then uncovered; the possible corrective movements of the previously covered eye are observed. If it moves: (a) outward, esophoria is present; (b) inward, exophoria; (c) downward, hyperphoria; and (d) upward, hypophoria. 

### The alternating cover test

This test is also useful to determine the maximum misalignment of the eye axes in both a tropia as well as a phoria. The alternating cover test is also helpful when establishing a vertical divergence/skew deviation (in the context of an OTR), i.e. a vertical misalignment of the eyes that cannot be explained by an ocular muscle palsy or damage to a peripheral nerve. In contrast to trochlear nerve palsy or superior oblique muscle palsy, in skew deviation as a component of the OTR the vertical misalignment changes little or not at all during different directions of gaze [12].

### Clinical examination with Frenzel’s glasses or a Fresnel-based device

**Fig. 2:**
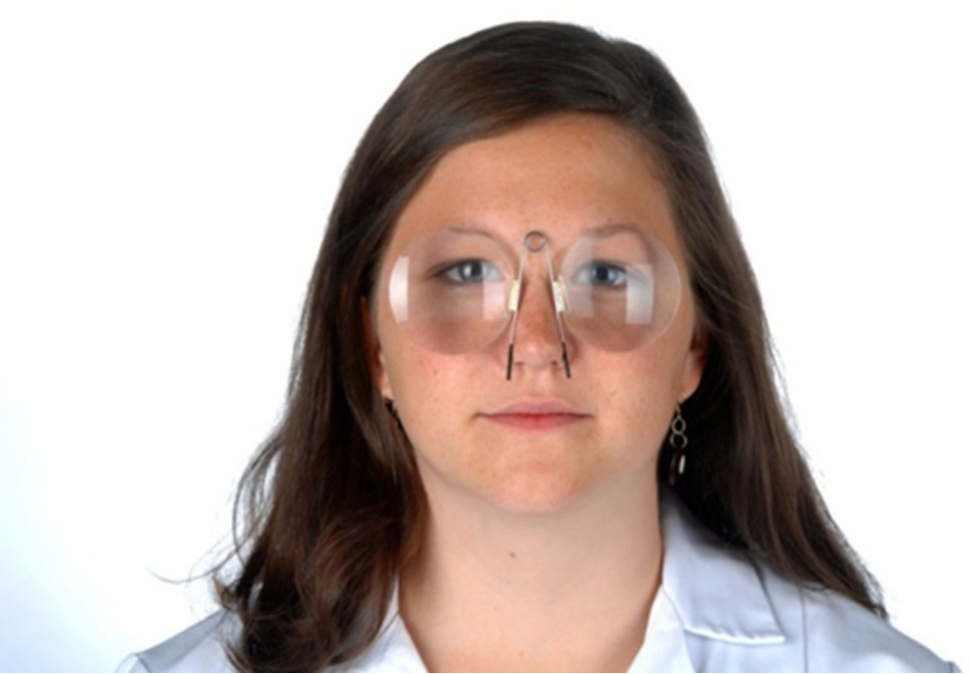
Clinical examination using a Fresnel-based device as an alternative to Frenzel’s goggles. The lenses prevent gaze fixation, which may suppress peripheral vestibular spontaneous nystagmus, for example. In addition, they make it easier to study the patient’s eye movements. When these lenses are used to examine a patient, attention should be paid to possible spontaneous nystagmus, GEN, head-shaking nystagmus (to this end, the patient should be asked to turn his/her head quickly from right to left and back, about 20 times; subsequently the eye movements should be studied), positional nystagmus, and hyperventilation-induced nystagmus. Positioning nystagmus indicates a muscle tonus imbalance of the VOR; if this originates from a peripheral vestibular lesion—as occurs, for example, in vestibular neuritis—then the nystagmus can be typically suppressed by visual fixation. Head-shaking nystagmus indicates a latent asymmetry of the so-called velocity storage; this may be due to peripheral or central vestibular functional disorders

The magnifying lenses (+16 diopters) with light inside, on the one hand, prevent visual fixation, which typically suppresses a peripheral vestibular spontaneous nystagmus, and on the other, facilitate the observation of the patient’s eye movements (Fig. 2). The examination should include peripheral vestibular spontaneous nystagmus, head-shaking nystagmus (for this test the patient is instructed to turn their head quickly to the right and to the left about 20 times; then the eye movements are observed), positioning and positional nystagmus, as well as hyperventilation-induced nystagmus. Spontaneous nystagmus indicates a tonus imbalance of the VOR. If it is caused by a peripheral vestibular lesion, as in vestibular neuritis, the nystagmus is typically dampened by visual fixation, whereas central fixation nystagmus is not suppressed by fixation or may become even worse. Head-shaking nystagmus is due to a latent asymmetry of the so-called velocity storage of the VOR, which can be due to peripheral and central vestibular disorders. In a peripheral vestibular deficit, the head-shaking nystagmus beats toward the ear with intact labyrinthine function. So-called cross-coupling can occur in central cerebellar disorders: the horizontal head-shaking maneuver induces vertical nystagmus (see [1]).

### Examination of the eye position in the nine eye positions

The position of the eyes should be examined when the patient is looking straight ahead in the eight eccentric positions to look for: (a) a range of eye movements and thereby positional deficits of one eye (e.g., in cases of paresis of the ocular muscle with a misalignment of the axes of the eyes) or both eyes (e.g., in progressive supranuclear gaze palsy (PSP)); (b) gaze-evoked nystagmus (GEN; i.e. disorders of the gaze-holding function, described below); and (c) nystagmus, whether its intensity changes depending on the direction of gaze (e.g., in DBN an increase in intensity when looking to the right, left and downward, or in peripheral vestibular spontaneous nystagmus an increase when looking in the direction of the quick phase and a decrease when looking in the opposite direction (‘Alexander’s law’).

**Fig. 3:**
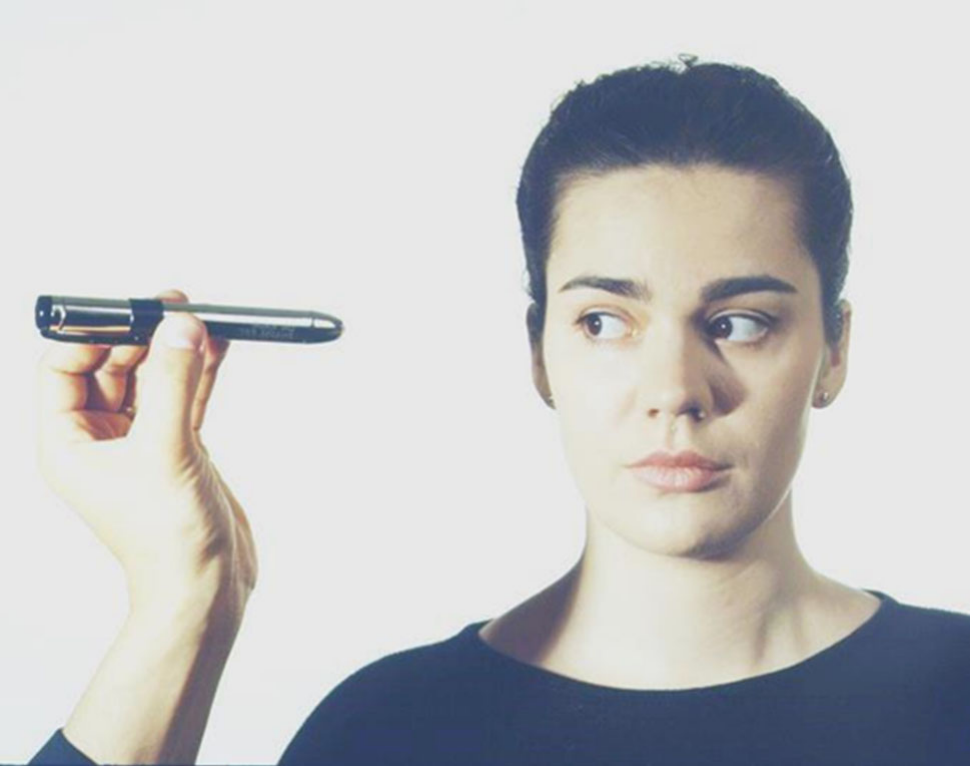
Clinical examination of eye position and eye movements with an examination flashlight. The advantage of this examination is that the images reflected on the retina can be observed and ocular misalignments identified. It is important that the examiner looks at the retinal images from the direction of the light and that the patient is instructed to fixate his/her gaze on the target object. GEN to all sides is usually caused by medication (such as antiepileptic drugs or benzodiazepines) or intoxication (e.g., alcohol). DBN increases when looking sideways and when looking downwards (modified from [3])

The examination can be performed with a small object for fixation or a small rod-shaped flashlight (Fig. 3). Using a small rod-shaped flashlight has the advantage that the corneal reflex images can be observed and thus ocular misalignments can easily be detected. It should be noted that it is important to observe the corneal reflex images from the direction of the illumination and to ensure that the patient attentively fixates the object. In the primary position one should first look for misalignment of the axes of the eyes and periodic eye movements, especially spontaneous nystagmus or so-called saccadic oscillations/intrusions. A nystagmus can be horizontal rotatory (typical for an acute vestibular neuritis), vertical downward or upward (DBN and UBN), or purely torsional. One should look for suppression of the nystagmus by visual fixation [typical for peripheral vestibular spontaneous nystagmus (see below)] or only slight suppression during fixation (or even an increase) of the intensity of the fixation (typical for central fixation nystagmus). Infantile/congenital nystagmus beats horizontally as a rule at various frequencies and amplitudes and increases during fixation.

Impaired visual fixation includes square-wave jerks (small saccades of 0.5°–5° with an inter-saccadic interval) which cause the eyes to oscillate around the primary position and are observed in progressive supranuclear palsy (PSP) or certain cerebellar syndromes. Other forms are ocular flutter (intermittent rapid bursts of horizontal oscillations without an inter-saccadic interval) or opsoclonus (combined horizontal, vertical and torsional oscillations also without an inter-saccadic interval) [13] which are not strictly forms of nystagmus but are so-called saccadic oscillations/intrusions. They occur in various disorders, for example, in brainstem encephalitis, tumors of the brainstem or cerebellum, intoxication, or most often in paraneoplastic syndromes.

### Examination for a gaze-holding deficit: GEN

The distinction between GEN (Fig. 3) and so-called end-point nystagmus is a widespread clinical problem. Many healthy subjects have physiological end-point nystagmus during maximal eccentric gaze. End-point nystagmus is pathological if it lasts for longer than 20 s (sustained end-point nystagmus), is notably asymmetrical, and/or is accompanied by other ocular motor disturbances [14].

GEN often allows a topographic anatomical diagnosis: (a) GEN in all directions occurs in cerebellar disorders, particularly impaired function of the flocculus/paraflocculus, and above all in neurodegenerative diseases, but can also be caused by drugs such as anticonvulsants, benzodiazepines or alcohol; (b) purely horizontal GEN can indicate a structural lesion in the area of the brainstem [nucleus prepositus hypoglossi, vestibular nuclei, and cerebellum (flocculus/paraflocculus)]—the neural integrator for horizontal gaze-holding function; (c) purely vertical GEN is observed in midbrain lesions involving the interstitial nucleus of Cajal (INC)—the neural integrator for vertical gaze-holding function; (d) dissociated horizontal GEN (greater in the abducting than the adducting eye) in combination with an adduction deficit is the sign of internuclear ophthalmoplegia (INO) due to a defect of the medial longitudinal fascicle (MLF), ipsilateral to the adduction deficit; (e) DBN usually increases when looking down, and especially to the side, most likely due to an additional gaze-holding deficit [15], so that the nystagmus beats diagonally downward in the sideward gaze (the cause of DBN is generally bilaterally impaired function of the flocculus/paraflocculus; (f) patients with GEN also often show a rebound nystagmus. To examine for so-called rebound nystagmus, the patient should gaze for at least 60 s to one side and then return the eyes to the primary position. This can cause a transient nystagmus to appear with slow phases in the direction of the previous eye position. Rebound nystagmus generally indicates damage to the flocculus/paraflocculus or cerebellar pathways.

### Clinical examination of smooth pursuit eye movements

The generation of smooth pursuit eye movements, which keep the image of an object stable on the fovea, involves diverse supra- and infratentorial structures: the visual cortex, medial temporal area, medial superior temporal area (MST), frontal eye fields (FEF), dorsolateral pontine nuclei, cerebellum (flocculus), and vestibular and ocular motor nuclei. These eye movements are influenced by alertness, a number of drugs and age. The patient is asked to track visually an object moving slowly in horizontal and vertical directions (10–20°/s) while keeping the head stationary. It is important that the subject is able to fixate the target. Corrective (catch-up or back-up) saccades are looked for; they indicate a smooth pursuit gain (ratio of eye movement velocity and gaze target velocity) that is too low or too high: a saccadic smooth pursuit in all directions indicates an impaired function of the flocculus/paraflocculus [e.g., in spinocerebellar ataxias, drug poisoning (anticonvulsants, benzodiazepines), or alcohol abuse]. However, marked asymmetries of smooth pursuit indicate a structural lesion. If the smooth pursuit is saccadic to the left, this indicates a left-sided lesion of the flocculus/paraflocculus.

### Clinical examination of saccades

**Fig. 4:**
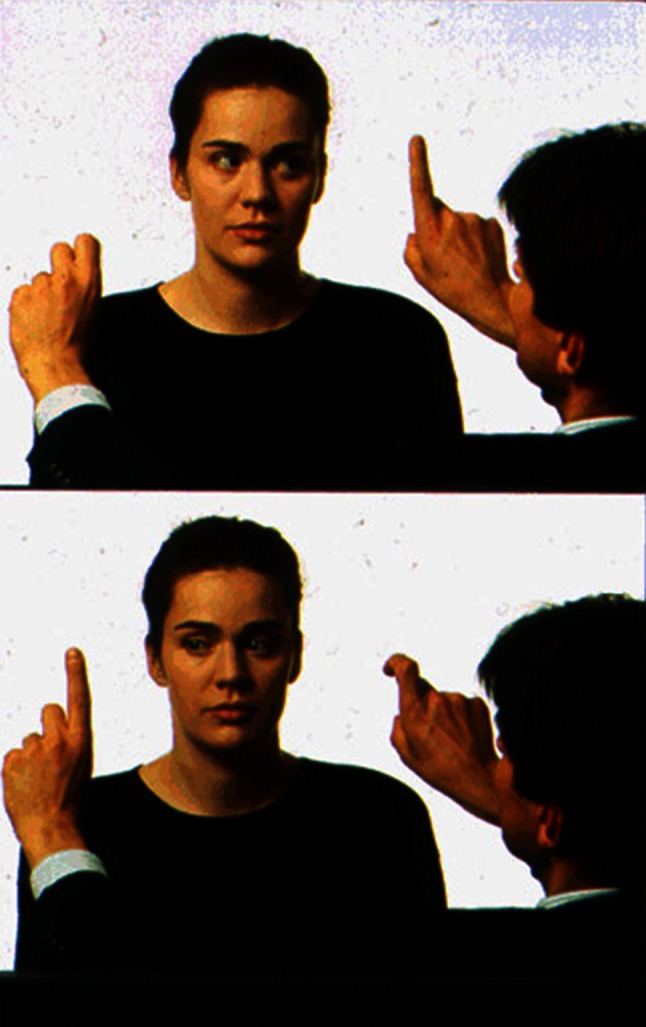
Clinical examination of saccades. Spontaneous saccades that are triggered by visual or acoustic stimuli should be studied first. Then the patient should be asked to switch his/her gaze between two horizontal and two vertical targets. The velocity and accuracy of the saccades should be observed, and whether they are conjugate. In healthy subjects, the target will be reached immediately or will be made by one correctional saccade. Slow saccades in all directions typically occur in neurodegenerative disorders. Slowed horizontal saccades are usually observed in pontine brainstem lesions and slowed vertical saccades in midbrain lesions. Hypermetric saccades, which are recognized by a corrective saccade back to the target, are found in cerebellar lesions. The pathognomonic sign of internuclear ophthalmoplegia is a slowed adducent saccade ipsilaterally to the defect of the medial longitudinal fasciculus. (Modified from [3])

**Fig. 5:**
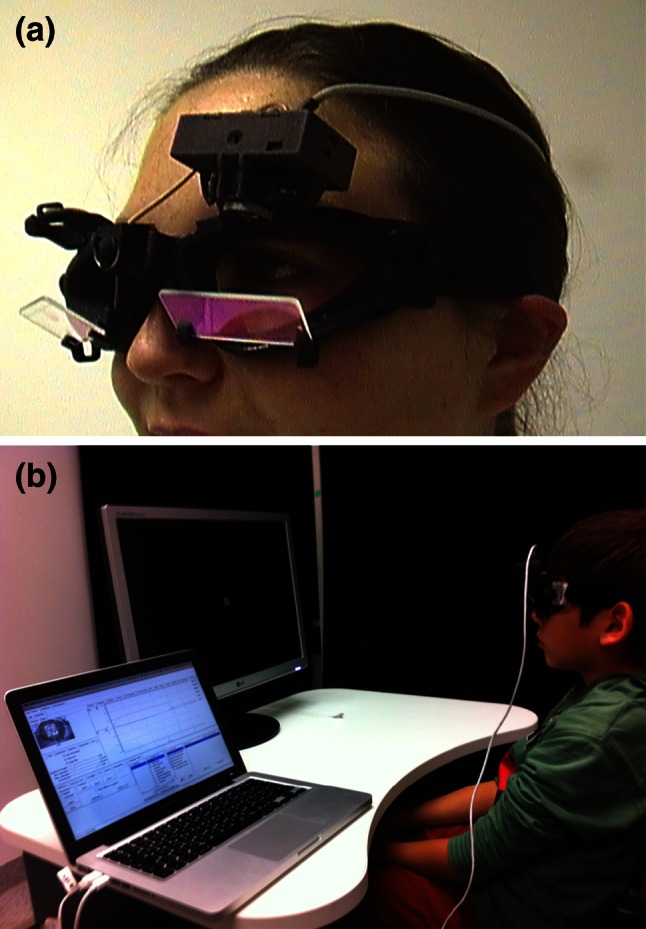
Video-oculography (VOG) allows the recording of all types of eye movements: **a** VOG device; **b** examination of a child, sitting in front of a screen, fixating and following the targets presented. VOG is particularly relevant to measuring the velocity of saccades to detect mild to moderate slowing as found in the initial stages of PSP or NP-C. Therefore, it could be a sensitive tool for an early diagnosis of these diseases

First, it is necessary to observe spontaneous saccades, for instance, when taking patient history and when triggered by visual or auditory stimuli. The patient is then asked to glance back and forth between two horizontal and two vertical targets (Fig. 4). The velocity, accuracy and the conjugacy of the saccades should be noted: (a) normal individuals can immediately reach the target with a single fast movement or one small corrective saccade; (b) slowing of saccades in all directions—often accompanied by hypometric saccades—occurs, for example, in neurodegenerative disorders or with intoxication (and with medication, particularly anticonvulsants and benzodiazepines); (c) isolated slowing of horizontal saccades is observed in pontine brainstem lesions due to a dysfunction of the ipsilateral paramedian pontine reticular formation (PPRF); this can be caused by ischemia, bleeding, pontine gliomas but also in Gaucher disease type 3, the later stages of NP-C and PSP; (d) isolated slowing of vertical saccades indicates a midbrain lesion in which the rostral interstitial medial longitudinal fascicle (riMLF) is involved, as in ischemic or neurodegenerative diseases, especially progressive supranuclear palsy or inherited disorders such as NP-C (in the latter, typically first downward and then downward and upward because there is a double innervation for upward saccades from the riMLF [16]—a bilateral lesion of the INC impairs the range of all types of vertical eye movements and is accompanied by a vertical GEN, and a lesion of the posterior commissure (PC) also impairs all types of vertical eye movements and is associated with a convergence-retraction nystagmus (see [1])); (e) hypermetric saccades, which can be identified by a corrective saccade back to the object, indicate lesions of the cerebellum (especially the vermis) or the cerebellar pathways. Patients with Wallenberg’s syndrome make hypermetric saccades toward the side of the lesion and hypometric saccades toward the opposite side due to a dysfunction of the inferior cerebellar peduncle (conversely, defects of the superior cerebellar peduncle lead to contralateral hypermetric saccades); (f) a slowing of the adducting saccade ipsilateral to a lesion of the MLF is pathognomonic for INO; (g) delayed initiation of saccades is most often due to supratentorial cortical dysfunction affecting the frontal or parietal eye field (e.g., Balint’s syndrome) and is called ocular motor apraxia. Nowadays, the velocity of saccades can be quantified in clinical routine by video-oculography (Fig. 5), which also allows the detection of mild to moderate slowing of saccades that could be the first clinical sign of PSP, NP-C or Gaucher disease type 3.

### Vergence test and convergence reaction

Vergence is tested by moving a target from a distance of about 50 cm toward the patient’s eyes or the patient looks back and forth between a distant and a near target. Looking at a nearby target causes vergence, accommodation and miosis (i.e. the convergence reaction). Neurons that are important for the convergence reaction are in the area of the mesencephalic reticular formation and the oculomotor nucleus. This explains why the convergence reaction is disturbed in rostral midbrain lesions and tumors of the pineal region and thalamus, and why abnormalities of vertical gaze are often associated with these defects. In certain neurodegenerative disorders such as PSP, convergence is also often impaired.

Inborn defects of the convergence reaction also occur in some forms of strabismus. Convergence-retraction nystagmus can be provoked by inducing upward saccades or by looking at a moving optokinetic drum with its stripes going downward. Instead of vertical saccades, rapid convergent eye movements that are associated with retractions of the eyeball occur. The cause is damage to the posterior commissure or, in rare cases, a bilateral disorder of the rostral interstitial nucleus of medial longitudinal fasciculus (riMLF). A spasm of the near reflex is a voluntary convergence accompanied by pupillary constriction. The latter is an important clinical sign for the diagnosis. Occasionally, spasm of the near reflex is psychogenic; it can mimic bilateral abducens palsy.

### Examination with the optokinetic drum

**Fig. 6:**
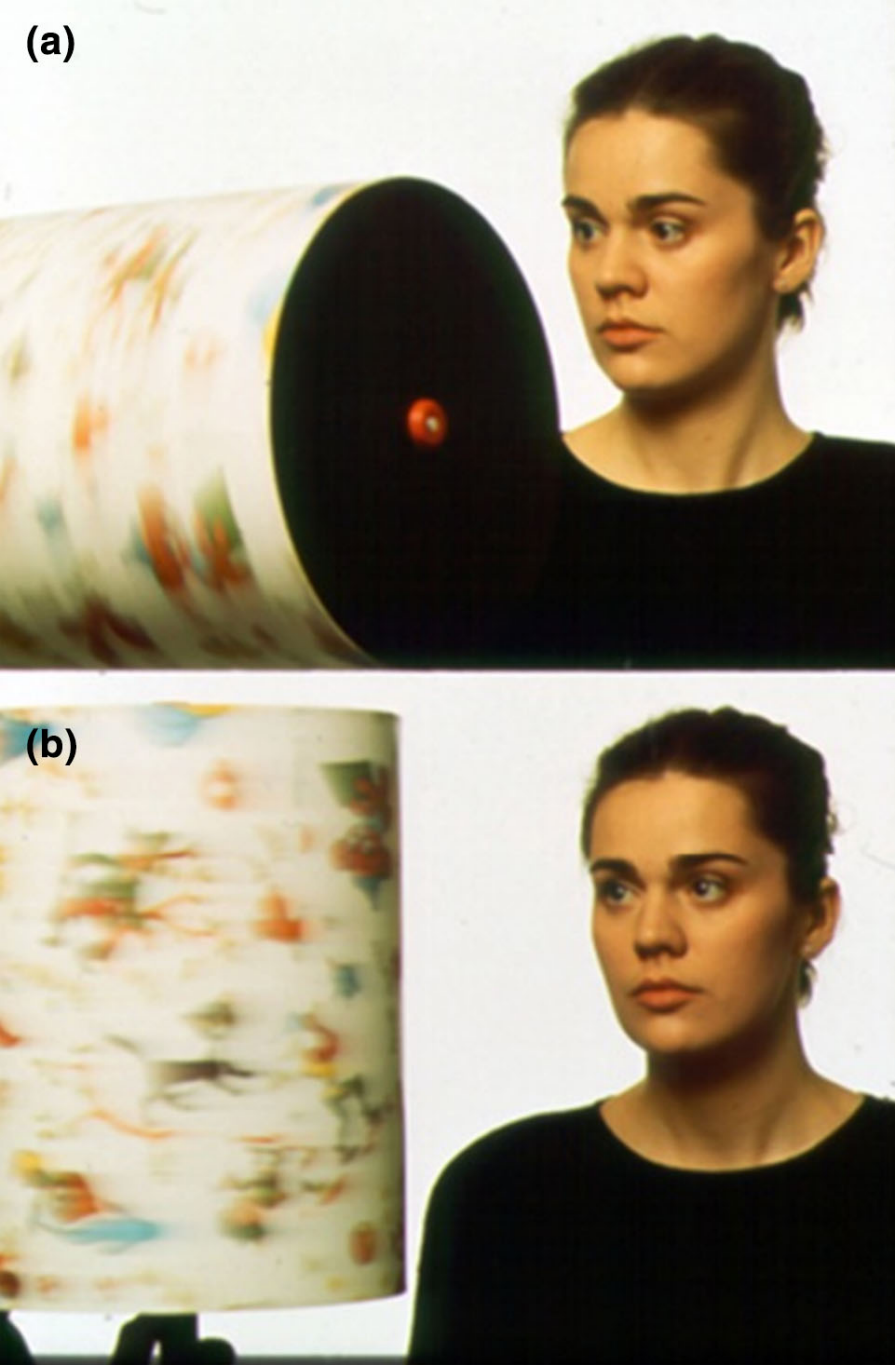
Examination of eye movements with an optokinetic drum: **a** vertical direction; **b** horizontal direction (modified from [3])

The examination of eye movements with the optokinetic drum allows combined testing of smooth pursuit movements and saccades in horizontal and vertical directions (Fig. 6). It is especially helpful with uncooperative or drowsy patients and with children. Intact horizontal and vertical optokinetic nystagmus probably indicates intact function of the midbrain and the pons. One should look for: asymmetries (e.g., between right and left (indicates a unilateral cortical or pontine lesion); vertical worse than horizontal (indicative of a vertical supranuclear gaze palsy due to a mesencephalic lesion); dissociations of the two eyes (a sign of diminished adduction in INO); and reversal of pursuit (indicates congenital nystagmus).

### The head impulse test: examination of the horizontal vestibulo–ocular reflex (VOR)

The most common bedside test of the examination of the VOR is the head impulse test, which examines the VOR at a high frequency [17]. To test the horizontal VOR the examiner holds the patient’s head between both hands, asks him to fixate a target in front of his eyes, and very rapidly turns the patient’s head horizontally approximately 20–30° to the right and then to the left [18]. This is the most important bedside test for VOR function. In a healthy subject this rotation of the head causes rapid, compensatory eye movements in the opposite direction with the same angular velocity as the head movements, so that the eye position in space remains the same. In this way the target also remains stable on the retina. For instance, in unilateral right-sided labyrinthine failure the eyes move during head rotations with the head to the right, and the patient has to perform a so-called re-fixation saccade to the left to fixate the target again. This is the clinical sign of a deficit of the VOR (in the high frequency range) to the right. If the findings of the bedside test are unclear, the use of a video-based head-impulse test is indicated; this allows the gain of the VOR to be quantified [19].

### Testing visual fixation suppression of the VOR

Before testing the visual fixation suppression of the VOR, the examiner must be sure that the VOR is intact (see above). The patient is then asked to fixate a target in front of his eyes while turning his head as uniformly as possible with the same angular velocity as the target in front of the eyes, first horizontally and then vertically, back and forth at moderate speed. The examiner should watch for corrective saccades, which indicate a disorder of the visual fixation suppression of the VOR. If the visual fixation suppression of the VOR is intact, the eye position relative to the head position does not change, but if it is not intact (which is indicated by small corrective saccades and as a rule occurs with smooth pursuit abnormalities, as these two functions use the same neural pathways) this typically indicates lesions of the cerebellum (flocculus or paraflocculus) or of cerebellar pathways [20]. Drugs, particularly anticonvulsants, sedatives and alcohol can also impair visual fixation suppression of the VOR because of their effects on the cerebellum. It is important to note that, in the case of a concomitant bilateral vestibulopathy, visual fixation suppression looks normal because the VOR is not working.

## Ocular motor disturbances

Topographically and anatomically, ocular motor disturbances can be classified as either peripheral or central. Peripheral forms affect the six outer and/or two inner ocular muscles or the oculomotor nerve, trochlear nerve or abducens nerve. Patients with peripheral ocular motor disturbances often complain of diplopia, which intensifies in the direction of the paretic muscle/nerve. Peripheral ocular motor disturbances usually affect one eye only (important exceptions include myasthenia gravis, chronic progressive, external ophthalmoplegia).

**Fig. 7:**
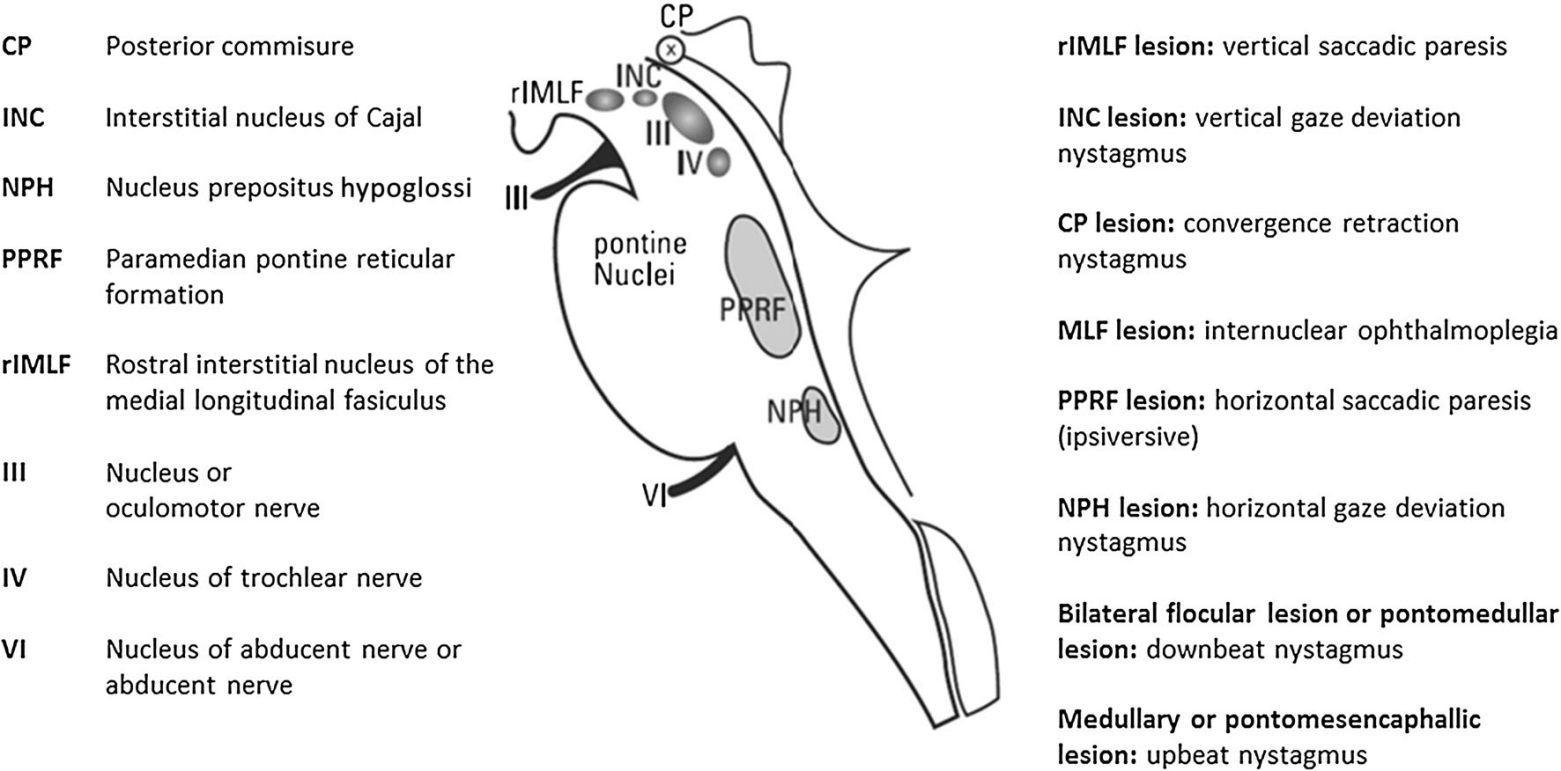
The supranuclear centers for control of eye movements. These centers allow exact topographical determination: lesions in the region of the interstitial nucleus of Cajal (INC) lead to a vertical gaze-holding defect; lesions in the region of the rostral interstitial nucleus of the medial longitudinal fasciculus (riMLF) lead to impairments of vertical saccades; lesions of the PPRF result in impairments of the horizontal saccades and; lesions of the nucleus prepositus hypoglossi (NPH) are characterized by a horizontal gaze-holding defect (adapted from [7, 37] and modified from [2])

Central forms usually affect both eyes. These are manifestations of functional impairments of the brainstem (Fig. 7), cerebellum, or (rarely) other higher level centers. Patients with central ocular motor disturbances may report unclear or blurred vision. If the ocular motor disturbance is slowly progressive, such as in PSP, cerebellar degeneration or NP-C, it may remain undetected for a long time. Usually, the extent of the subjective impairments also depends on how acutely the impairments develop.

Central ocular motor disturbances can be classified as follows:

- *Fascicular lesions*—defects of the short part of the individual ocular muscle nerves within the brainstem. These are very rare; at first glance they look like unilateral peripheral lesions, but are accompanied by central ocular motor disturbances.
- *Nuclear lesions*—defects of the oculomotor nucleus (because of the anatomical proximity, almost always both nuclei are affected), the trochlear nucleus, or the abducens nucleus.
- *Supranuclear lesions*—due to defects of ocular motor pathway systems or supranuclear nuclei (Table 2a). Supranuclear ocular motor disturbances usually impair the movement of both eyes, for example, in the form of gaze palsy, slowed saccades, saccadic pursuit, or a gaze-holding defect, because the structures that are higher level to the cerebral nerve nuclei are affected. Often, such ocular motor disturbances are associated with other neurological deficits, so that the ‘overlap’ of the neurological findings allows location of the level of the lesion in the brainstem region as well as the side.

**Table 2:** Overview of the (a) anatomical origin of ocular motor disturbances and nystagmus and (b) of the functional anatomy of the cerebellum with regard to ocular motor disturbances and nystagmus (modified from [2])

Clinical finding: oculomotor disturbances and nystagmus	Suspected location of damage in the brainstem/cerebellum
(a) Anatomical origin of oculomotor disturbances and nystagmus	
Isolated vertical saccadic paresis	Midbrain (riMLF)
Isolated horizontal saccadic paresis, isolated unilateral horizontal saccadic paresis	Pons (PPRF) lesion ipsilateral to PPRF
Hypermetric saccades	Cerebellum (fastigial nucleus; also in Wallenberg syndrome (toward the side of the lesion) affecting cerebellar pathways)
Isolated vertical gaze-evoked nystagmus (up and down)	Midbrain (INC, the neuronal integrator of vertical [and torsional] eye movements)
Isolated gaze-evoked nystagmus (right and left)	Pontomedullary/cerebellar (nucleus prepositus hypoglossi (NPH), vestibular nuclei, vestibule-cerebellum [of the neuronal integrator of horizontal eye movements])
Internuclear ophthalmoplegia (INO)	Ipsilateral MLF (lesion on the side of impaired eye adduction)
Downbeat nystagmus (DBN)	Mostly cerebellum with bilateral flocculus impairment
Upbeat nystagmus	Medulla oblongata or midbrain
Convergence-retraction nystagmus	Midbrain (posterior commissure)

Location of damage	Typical findings
(b) Functional anatomy of the cerebellum with regard to oculomotor disturbances and nystagmus	
Flocculus/paraflocculus	Saccadic pursuit, downbeat nystagmus, rebound nystagmus, impaired visual fixation of the VOR
Nodulus/uvula	Central positional nystagmus, periodic alternating nystagmus
Vermis/fastigial nucleus	Hypometric (dorsal vermis) or hypermetric (fastigial nucleus) saccades

*DBN* downbeat nystagmus, *INC* interstitial nucleus of Cajal, *MLF* medial longitudinal fasciculus, *PPRF* paramedian pontine reticular formation, *riMLF* rostral interstitial nucleus of the medial longitudinal fasciculus, *VOR* vestibulo-ocular reflex
- *Cerebellar impairments*—lead to impaired smooth pursuit, gaze-holding function, or saccades (Table 2b).

## Topographical anatomy

Only a few brainstem centers, which have clearly allocated functions, are important for triggering and controlling eye movements (Fig. 6; Table 2a). This makes their pathological anatomy easy to understand. The following simple clinical rule applies: horizontal eye movements are generated and controlled in the pontine region, whereas vertical and torsional eye movements originate in the midbrain.

### Midbrain centers

The center for vertical saccades is the rostral interstitial nucleus of the medial longitudinal fasciculus (riMLF), and the center for vertical gaze-holding function (the vertical and torsional neural integrator) is the interstitial nucleus of Cajal (INC). It is important to note that a normal floccular function is also required for gaze holding. Clinically, this means that an isolated vertical saccadic paresis or isolated vertical gaze deviation nystagmus would suggest a midbrain lesion.

### Pontine and pontomedullary centers

The center for horizontal saccades is the paramedian pontine reticular formation (PPRF); clinically this means that isolated horizontal saccadic palsy indicates a pontine lesion, and a unilateral PPRF lesion will result in saccadic disturbances on the side of the lesion. The center for the horizontal gaze-holding function is the nucleus prepositus hypoglossi together with the vestibular nuclei and the vestibulocerebellum (the horizontal neuronal integrator). Purely horizontal GEN originates from a pontine lesion.

### Cerebellar centers

Cerebellar lesions are often accompanied by easily clinically identifiable ocular motor disturbances. For example, defects of the flocculus/paraflocculus are characterized by saccadic pursuit, DBN, and impairments of the visual fixation suppression of the VOR (Table 2b). Lesions of the ocular motor vermis (Lobulus VII) and the fastigial nucleus lead to saccadic dysmetria, whereas nodulus/uvula lesions can induce periodic alternating nystagmus. Paraneoplastic cerebellar disorders often lead to opsoclonus (see above) in addition to the ocular motor disturbances mentioned above.

## Eye movement disorders in neurodegenerative diseases

Central ocular motor disorders are one of the key symptoms in a broad spectrum of inherited or acquired neurological and systemic disorders. Some of them are now treatable and will be described below in more detail along with the most important differential diagnoses. Since a review on the impairment of eye movements was published on PSP [21], this article will not deal with PSP in detail but will focus on treatable diseases and spinocerebellar ataxias.

### Niemann–Pick disease type C

Niemann–Pick disease type C (NP-C) is an autosomal recessive neurovisceral disorder caused by mutations in either the *NPC1* or the *NPC2* gene (see [22]). The estimated disease incidence is 1:120,000 live births, but may be higher, as the disease is probably under-diagnosed due to its heterogeneous presentation [23]. NP-C is characterized by visceral, neurological and psychiatric manifestations that are not specific to the disease as they are often observed in other diseases. Sufferers accumulate massive amounts of cholesterol and other lipids in the late endosome/lysosomal compartment caused by a defect in intracellular lipid trafficking; cholesterol accumulation mainly occurs in the peripheral organs, while glycosphingolipids principally accumulate the central nervous system. These alterations in lipid storage lead to organ enlargement cell functional impairment, with subsequent cell loss [24, 25].

**Fig. 8:**
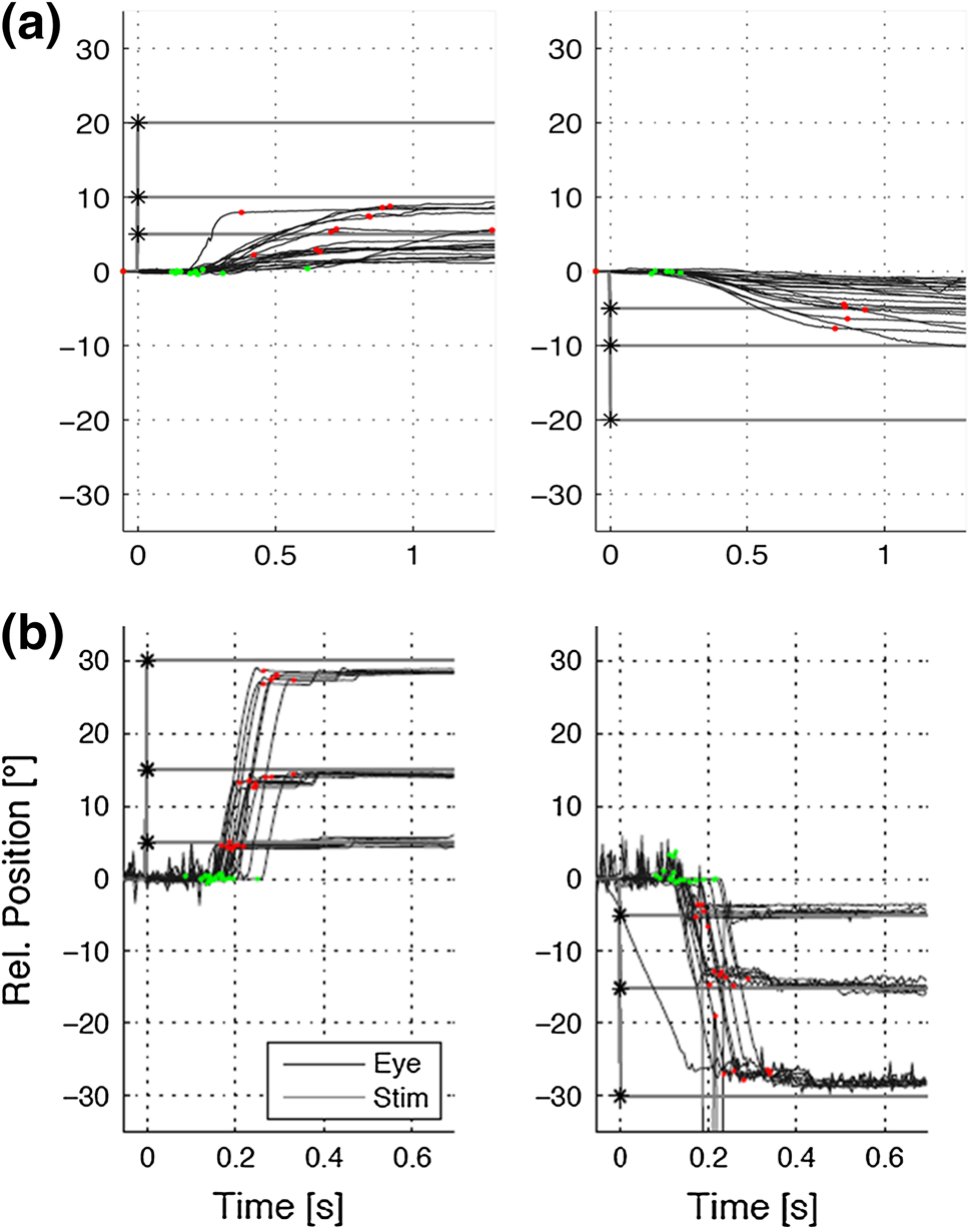
Recording of: **a** vertical and **b** horizontal saccades in a patient with NP-C. There is a significant slowing of the velocity of vertical saccades (upward 26°/s, downward 11°/s). The velocity of horizontal saccades is more than 350°/s which is within the lower normal range. Typically, there is first a slowing of vertical downward, then upward and finally also horizontal saccades in patients with NP-C

Vertical saccade paresis (Fig. 8) may be present before the internal, neurological or psychiatric manifestation and is sometimes the only symptom of NP-C in adults [26], thus representing a ‘red flag’ signaling the need for further diagnostic work-up. Initially, slow vertical saccades, especially downwards, often accompanied by horizontal oscillations (as an expression of activation of the intact horizontal saccade system) and frequent blinking occur. The other ocular motor systems may also be affected to varying degrees. Vertical optokinetic nystagmus, as tested by an optokinetic drum, is often absent. At the beginning of the disease, smooth pursuit might be intact. As the disease progresses, complete vertical gaze palsy with the inability to look upward or downward occurs [27–29]. The VOR is often preserved until very late, indicating that the gaze palsy is truly supranuclear in nature [16]. The pathological vertical saccades, which are the first indications of this disease, may be overlooked in a superficial examination due to the initial discrepancy between smooth pursuit and saccades. Therefore, a detailed and careful neuro-ophthalmological examination is crucial for the diagnosis of inherited disorders.

Early identification of NP-C and the appropriate application of symptomatic and disease-specific therapies can dramatically improve the quality of life of these patients. Visceral manifestations comprise neonatal jaundice during infancy, present in 39 % of patients; hepatomegaly during infancy in 39 %; and splenomegaly in 54 % [30]. However, in adulthood, hepatosplenomegaly may be very mild and is not always present. Neurologically, the disease manifests with clumsiness and frequent falls as an expression of stance and gait ataxia, changes in muscle tone leading to dystonia, myoclonus, epilepsy, dysarthria, and dysphagia. Cognitive impairment is one of the most consistent neurological findings, reported in 60–70 % of patients across all age-at-onset categories. Impaired cognition frequently manifests in poor school performance in juveniles and adolescents and, as NP-C progresses, patients experience a general decline leading to dementia in many cases. In younger-onset patients, NP-C is more likely to be identified initially as developmental delay [30]. So-called gelastic cataplexy—a sudden loss of muscle tone especially in emotional situations such as laughing or crying—is a pathognomonic symptom that is sometimes present in children.

Establishing a diagnosis of NP-C in adults can be difficult as the disease may only manifest psychiatrically in the form of schizophrenia-like psychosis, but psychiatric disorders such as depression or bipolar disorder have also been described [31]. The diagnostic algorithm encompasses biochemical examination of plasma chitotriosidase activity and oxysterol levels, as well as filipin staining of patient skin fibroblasts, molecular-genetic examination by *NPC1* and *NPC2* gene sequencing, and abdominal ultrasonography [32].

Therapy with miglustat 200 mg t.i.d. has been shown to stabilize the neurological manifestations of the disease, and it has been suggested that early therapy in affected children may halt or slow neurological disease progression [32–35].

### Gaucher disease type 3

Gaucher disease (GD) is an autosomal recessive lysosomal storage disorder caused by the absence of the enzyme glucocerebrosidase, leading to accumulation of glucocerebroside in tissue macrophages [36]. GD is classified as GD1 (non-neuronopathic), GD2 (acute neuronopathic), and GD3 (chronic neuronopathic) depending on the presence of neurological deterioration, age at identification and disease progression rate. The chronic neuronopathic form (GD3) may be divided into three subtypes: type 3a has a fulminant neurological course complicated by myoclonic seizures but with mild visceral involvement; type 3b has severe visceral symptoms and signs such as massive hepatosplenomegaly, moderate to severe kyphosis, but no neurological involvement; and type 3c is characterized by mild visceral involvement, mild kyphosis and life-threatening progressive heart valve calcification.

GD3 is associated with a severe slowing of the horizontal saccades, which leads progressively to horizontal supranuclear palsy with impairment of all horizontal eye movements including smooth pursuit and the VOR [37]. To compensate for the deficits of the horizontal saccades, patients perform torsional saccades as an expression of compensation by the initially intact vertical saccade system. The other compensatory mechanism is the VOR: the head is quickly rotated to the contralateral side, bringing the eyes to the desired position. This manifests clinically as fast thrusting head movements (differential diagnosis: Cogan syndrome, see below). As the disease progresses, a vertical saccade palsy may also develop.

Testing of enzyme activity in leukocytes, genetic testing, and abdominal ultrasonography represent a clinical standard to establish the diagnosis of GD. Disease-specific enzyme replacement therapy (ERT) with intravenous infusions of the enzyme imiglucerase 5–60 IU/kg for 2 weeks, or alternatively taliglucerase or velaglucerase alfa improves disease symptoms and outcomes in patients with non-neuronopathic Gaucher disease type 1, but published data on the treatment of GD3 are very limited, primarily because these large protein-based therapies do not cross the blood–brain barrier [38]. The efficacy of the oral substrate reduction therapy (SRT), miglustat, in GD3 has been evaluated in one randomized, controlled clinical trial as miglustat is a small molecule that is able to access the CNS, but findings were inconclusive with regard to beneficial effects on neurological symptoms [39]. A second oral SRT, eliglustat, is currently under clinical development but has been shown not to cross the blood–brain barrier and, as with ERT, is therefore not expected to be of therapeutic use for neuronopathic GD [40]. Treatment with the chaperone, ambroxol, has also been shown to restore enzyme activity in vivo, leading to clinical improvements [41].

### Tay–Sachs disease

Tay–Sachs disease (TS) is an autosomal recessive, predominantly cerebellar disease of the sphingolipid metabolism caused by a beta-hexosaminidase A deficiency with a subsequent accumulation of GM2 gangliosides in organ systems including the central nervous system. The disease manifests in early childhood (in the case of homozygotes with full enzyme deficiency), although in heterozygotes (normally G269S mutation) with residual beta-hexosaminidase A activity (late-onset Tay–Sachs [LOTS]), the first disease symptoms occur in adolescence (mean age 18.1 years) [42]. Clinical presentations feature a range of neurological signs that are not specific to the disease, with the functional impairment of primary motor neurons expressed in spastic stance and gait combined with secondary motor neuron impairment, leading to muscle atrophies with fasciculations and consecutive fatigue. Furthermore, patients present with cerebellar ataxia with postural instability, and extrapyramidal symptoms. Psychiatric disorders, including psychotic episodes, depression, and cognitive decline, may occur as part of the clinical picture [43, 44]. Previous MRI studies have shown cerebellar atrophy as a morphological correlate [43].

In terms of ocular motor deficits, patients demonstrate hypometric saccades with a distinctive saccade abnormality consisting of abrupt fluctuations in saccade velocity with premature termination (in general, velocity does not decrease below 50°/s). In general, saccades stop sooner and faster in LOTS patients. Moreover, lower smooth pursuit gain and a reduction of the slow phase of the optokinetic nystagmus may occur. Currently, there is no disease-specific therapy. Treatment with pyrimethamine, up to a maximum of 75 mg/day, which is believed to increase hexosaminidase A activity, has induced a discrete improvement of dysarthria, dysthymia and fall frequency [45].

## Differential diagnoses

### Cogan syndrome or infantile saccade initiation delay syndrome (ISID)

ISID is characterized by the inability to initiate saccades on command [46]. Saccades are prolonged and hypometric, and reflexive saccades, including the quick optokinetic and VOR phases, may also be impaired [47]. In contrast, saccadic velocity is normal. Head thrust and thereby the VOR act as a compensatory mechanism using the VOR to initiate saccades, which is present in 85 % of patients, and is characteristic of this disease. Blinking without the VOR initiates saccades in 41 % of cases reported to date [47]. The saccadic impairment is not isolated, as smooth pursuit is impaired in around 33 % of cases. This, in combination with the reported developmental delay, hypotonia and speech disturbances in these children, indicates a complex impairment of the central nervous system. The term ‘ocular motor apraxia’ should not be used, because saccades on command and reflexive saccades are disturbed. In apraxia, reflexive saccades should be intact.

### Ocular motor apraxia type I (AOA1)

AOA1 is an autosomal recessive disease characterized in all cases by the presence of cerebellar ataxia, cognitive impairment, and high-grade sensorimotor polyneuropathy. In some cases, AOA1 is also associated with ocular motor apraxia (86 %), hypoalbuminemia, and hypercholesterolemia. The disease manifests between 2 and 18 years (mean age at onset 6.8 years). Saccades are prolonged and may imitate slow saccades but, in fact, hypometric saccades are elicited [48].

### Ocular motor apraxia type 2

Ocular motor apraxia type 2 (AOA2) is an autosomal recessive cerebellar ataxia with a typical age at onset between 3 and 30 years, and characterized by high-grade axonal sensorimotor neuropathy, ocular motor apraxia and a high alpha-fetoprotein concentration. Saccades are hypometric with a typical staircase pattern. The VOR in the form of head thrusts may be used as a compensatory mechanism [49, 50].

### Spinocerebellar ataxia type 2

Spinocerebellar ataxia type 2 (SCA 2) belongs to the family of autosomal dominant diseases. SCA 1, 2, 3, 6, 7 and 17 and dentatorubral-pallidoluysian atrophy are caused by an expansion of a polyglutamine (polyQ)-coding CAG triplet within the respective genes [51, 52]. SCA 2 is a ‘cerebellar plus’ syndrome which manifests with cerebellar ataxia with postural instability, uncoordinated stance and gait, dysmetria, dysarthria, dysphagia and dysdiadochokinesia [53]. Moreover, an l-Dopa-responsive akinetic-rigid syndrome might be present in some cases. Retinitis pigmentosa (gradual loss of retinal epithelium with initial night blindness, and progression to total blindness; pigmentation of the retina is typical) and myoclonic epilepsy could be part of the clinical picture. Moreover, patients show an action and postural tremor, peripheral neuropathy with early-onset areflexia of the upper extremities, executive dysfunction and cognitive decline.

Regarding eye movements, patients with SCA 2 exhibit early and dominant slowness of the horizontal saccades up to complete saccade paresis; vertical saccades are also impaired [54]. One prospective ocular motor study found that absence of GEN or dysmetric saccades in these patients has a high negative predictive value for the presence of SCA 2 [55]. Proper saccade generation is necessary to evoke GEN, and for performance of correcting hypo-/hypermetric saccades, while in the case of saccade impairment, the correcting saccades cannot be performed. It is not possible to distinguish between different types of spinocerebellar ataxia on the basis of the ocular motor examination. However, in some cases, such as in SCA 3, saccadic intrusions as well as saccadic oscillations in the form of square-wave jerks (short eye movements with an inter-saccadic interval), or ocular flutter (horizontal oscillations without an inter-saccadic interval) help to establish the diagnosis with targeted genetic testing. DBN, vertical positional nystagmus, as seen in positioning maneuvers, and abnormal head-shaking nystagmus (vertical nystagmus elicited by horizontal head shaking) are pathognomonic for SCA 6.

### Spinocerebellar ataxia type 7

Spinocerebellar ataxia type 7 (SCA 7) is characterized by severe cerebellar atrophy and retinal dystrophy with initial yellow–blue blindness, which usually progresses to total blindness due to retinitis pigmentosa. The neurological manifestation comprises high-grade ataxia and dysarthrophonia, dysphagia, pyramidal signs, pathological somatosensory responses (pathological somatosensory evoked potentials in terms of sensory-axonal polyneuropathy), pathological acoustic evoked potentials (AEP), or pathological visual evoked potentials (VEP) with P100 wave loss.

Anatomical studies have shown severe cerebellar degeneration and region-specific neocortical atrophy in SCA 7 patients [56]. Functional imaging has shown reduced functional interaction between the cerebellum and the middle and superior frontal gyri, and disrupted functional connectivity between the visual and motor cortices compared with healthy controls. With respect to eye movements, patients with SCA 7 have slow (especially horizontal) saccades. However, other ocular motor signs are unspecific and allow no specific differentiation of this disease from the other types of cerebellar ataxia [52]. Therapy of ataxia is symptomatic as no disease-specific therapy exists. For the treatment of different types of cerebellar ataxias, a positive effect of acetyl-dl-leucine 5 g/day on stance and gait stability, fine motor skills, and tremor was demonstrated in terms of an improvement in cerebellar ataxia rating scores was described [57]. Therapy with the potassium channel blocker, 4-aminopyridine, at doses of 5 mg t.i.d. under regular ECG checks (a prolonged QT is a possible side effect) might be considered to treat the ocular motor abnormalities, especially if DBN is present (see [58]). If diplopia is present, prism goggles might be used.

### Mitochondrial diseases: chronic progressive external ophthalmoplegia and Kearn–Sayre syndrome

Mutations in mitochondrial DNA (mtDNA) lead to multisystem symptomatology with a heterogonous manifestation dependent on the organ system and the number of mutated mitochondria in cells [59]. If all the mtDNA molecules present in a cell are identical (all wild type or all carrying a mutation), this condition is known as homoplasmy. When mtDNA with different sequences (pathogenic or not) are present in a single cell, the condition is known as heteroplasmy. The latter is common for pathogenic mutations, as only a portion of the cellular mtDNA content is affected. Heteroplasmy is a major factor that determines the clinical severity of mitochondrial diseases because mitochondrial function only begins to be affected when there is a relatively high number of mutated mtDNA compared to wild type, usually N > 70–80 % [15]. This phenomenon is known as the ‘threshold effect’ and it can vary depending on the mutation, the cell type, the tissue or even the affected individual. Heteroplasmy can be dynamic, changing during the lifetime in both mitotic and post-mitotic tissues due to cell cycle-independent mtDNA replication.

Chronic progressive external ophthalmoplegia (CPEO) begins in young adulthood (mean age 17.5 years without further symptoms) [59]. The cardinal symptom is conjugated horizontal and vertical gaze palsy (symmetrical impairment of external eye muscles) [59]. Initially, horizontal as well as vertical saccades are very slow; the saccade latency is prolonged. Patients suffering from diplopia or oscillopsia [60] present with asymmetrically affected eye muscles when the eye axes are not aligned. The disease progresses, as the name suggests, to complete ophthalmoplegia; ‘staring eyes’ with no possible eye movement are typical. The gaze palsy is associated with a bilateral, usually symmetrical ptosis. Gains of VOR and smooth pursuit are also reduced [61]. CPEO is often isolated and represents the mild variant of complex mitochondrial disorder. When other symptoms are also present (e.g., weakness of the oropharyngeal muscles with related speech and swallowing difficulties), it is referred to as CPEO plus syndrome. Moreover, weakness of proximal muscles and muscle cramps occurs during physical activity. Volumetric brain measurements revealed cortical and cerebellar atrophy as a morphological correlate of ocular motor disturbances.

Progressive external ophthalmoplegia is a part of Kearn–Sayre syndrome, a multisystem disorder with central nervous system involvement caused by a large-scale mtDNA deletion. Onset before 20 years of age, cerebellar ataxia, pigmentary retinopathy (usually rod-cone dystrophy), and in some cases, cardiac conduction block (usually the cause of death in young adulthood) and elevated cerebrospinal fluid protein level are typical for this syndrome [62]. Other neurological problems may include proximal myopathy, exercise intolerance, ptosis, oropharyngeal and esophageal dysfunction, sensorineural hearing loss, dementia, and choroid plexus dysfunction resulting in cerebral folate deficiency. Moreover, CPEO may be present in MELAS syndrome (mitochondrial myopathy, encephalopathy, lactate acidosis and stroke-like episodes), MERRF syndrome (myoclonic epilepsy with ragged red fibers), or ARCO syndrome (autosomal recessive cardiomyopathy, ophthalmoplegia).

### Whipple’s disease

Whipple’s disease (WD) is a rare chronic poly-systemic infection by a Gram-positive bacillus, *Tropheryma whipplei* (*T. whipplei*) and represents an important differential diagnosis for PSP. Ocular motor symptoms include initial vertical gaze palsy, with later horizontal gaze palsy and the picture of complete ophthalmoplegia (with impaired saccade and smooth pursuit systems). This symptomatology may develop within months. In PSP patients, horizontal saccades are usually hypometric and slow, but complete horizontal gaze palsy is rather rare. Moreover, PSP patients develop square-wave jerks, which are absent in WD patients. In a study with 18 WD patients [63], 17 % developed complete ophthalmoplegia. Part of the clinical picture is a systemic symptomatology, such as gastrointestinal symptoms, weight loss and, in the majority of cases, transient, recurrent and roughly symmetric polyarthralgia or nonerosive polyarthritis.

The neurological manifestations of the disease are diverse and can mimic almost any neurological condition. They include cerebellar ataxia and extrapyramidal signs presented in a different pattern than in PSP: absence of rigidity and normal gait and balance functions are typical for WD patients. Oculomasticatory myorhythmia (eye pendular vergence oscillations with a frequency of 1 Hz and concurrent contractions of the masticatory muscles) is rare, but pathognomonic [64]. Moreover, agrypnia excitata, a condition of severely reduced or absent sleep due to organic disorders with generalized motor and autonomic hyperactivation related to dysfunction in the thalamo-limbic circuits, occurs. Tremor, postural instability, dystonia, myoclonus, cognitive deficits, and delirium, progressing to coma and epileptic seizures may also be present. The diagnosis can be confirmed by the polymerase-chain reaction of all body samples as well as by immunohistochemistry methods. Therapeutically, treatment with trimethoprim–sulfamethoxazole and—in complicated cases—in combination with third-generation cephalosporins or doxycycline is indicated [65].

## Common forms of central nystagmus

Finally, we describe the two most common forms of nystagmus and the current therapeutic approaches: DBN and UBN [66], and their treatment with aminopyridines [58, 67, 68]. Both are types of fixation nystagmus that, in contrast to other types of peripheral vestibular spontaneous nystagmus, can hardly be suppressed by gaze fixation, which instead increases them, leading to blurred vision and oscillopsia.

### Downbeat nystagmus

DBN is the most common form of persistent nystagmus. It is a type of fixation nystagmus with the fast-phase beating in a downward direction. It generally increases when looking to the side and down and when lying prone. DBN manifests in 80 % of patients with uncertain posture and gait and in 40 % with vertical oscillopsia [66], and is usually due to a bilateral defect of the cerebellar flocculus [69]. The causes are degenerative disorders of the cerebellum, cerebellar ischemia, or Arnold–Chiari malformation. In some cases, it can be caused by paramedian lesions of the medulla oblongata.

The defect of the flocculus will result in reduced release of gamma-aminobutyric acid (GABA) and thus, the disinhibition of the vestibular nuclei. On the basis of this pathological mechanism, a prospective, randomized, placebo-controlled study of the effects of aminopyridines has indicated a significant improvement of symptoms [68], and the observed effects have since been supported by findings from other studies [70, 71]. The strongest effect was observed in patients with cerebellar atrophy [63]. Currently, 4-aminopyridine 2 × 5–2 × 10 mg/day is recommended off-label for therapy. However, such off-label medication should always be undertaken based on clinicians’ judgment of potential risks versus benefit, and with a control ECG performed 1 h before and after the first ingestion of the drug. The QTc interval should not be prolonged. Since the drug only has a symptomatic effect, continuous treatment is required. The stipulated mechanism of action is an increase in the resting activity and excitability of Purkinje cells; this was confirmed by in vitro studies [72]. Animal studies have also shown that aminopyridines synchronize the irregular spontaneous activity of Purkinje cells [73]. By means of an increased release of GABA, this is assumed to strengthen the inhibitory influence of Purkinje cells on vestibular/cerebellar nuclei.

### Upbeat nystagmus

Upbeat nystagmus (UBN) is rarer than DBN and is also a fixation nystagmus. In primary position, the UBN beats upward. Oscillopsias are often very irritating, but the symptoms are usually transient. In most cases, paramedian lesions in the medulla oblongata or the midbrain are found, for example, in patients with multiple sclerosis, brainstem ischemia or tumors, or Wernicke’s encephalopathy [19]. Observational studies have shown a positive effect of baclofen (15–30 mg/day) [74] and 4-aminopyridine (5–10 mg/day) [75].

## Conclusions

The clinical examination of the different eye movements (pursuit, saccades, gaze holding function) and nystagmus (spontaneous nystagmus or fixation nystagmus) allows topographic-anatomic diagnosis in the brainstem or cerebellum and differentiation between peripheral and central ocular motor and peripheral and central vestibular lesions in most cases. Isolated impairments of vertical eye movements (for example, impaired vertical saccades, vertical gaze palsy, isolated vertical gaze-evoked nystagmus) indicate a lesion in the midbrain, while isolated impairments of horizontal eye movements indicate a lesion in the pons. Cerebellar impairments can result in a multitude of ocular motor disturbances, such as saccadic pursuit, gaze-evoked nystagmus in all directions, impaired visual fixation suppression of the VOR, or DBN.

Ocular motor abnormalities can serve as vital diagnostic clues across a wide range of progressive neurological conditions as well as in acute central disorders. Appropriate ocular motor evaluations are particularly important among the neurodegenerative metabolic diseases, which can often be difficult to detect and diagnose at an early stage. For instance, saccadic eye movement abnormalities are often the first visible neurological sign in NP-C, and should prompt further, multidisciplinary diagnostic work up. As treatment options currently exist for some chronic, degenerative disorders associated with ocular abnormalities (e.g., miglustat for NP-C and aminopyridines for DBN and UBN) it is particularly important to identify the underlying condition as early as possible to initiate treatment.

## References

[1] Leigh RJ, Zee D (2006). The neurology of eye movements.

[2] Strupp M, Hufner K, Sandmann R, Zwergal A, Dieterich M, Jahn K, Brandt T (2011). Central oculomotor disturbances and nystagmus: a window into the brainstem and cerebellum. Dtsch Arztebl Int.

[3] Brandt T, Dieterich M, Strupp M (2013). Vertigo and dizziness—common complaints.

[4] Cnyrim CD, Newman-Toker D, Karch C, Brandt T, Strupp M (2008). Bedside differentiation of vestibular neuritis from central “vestibular pseudoneuritis”. J Neurol Neurosurg Psychiatry.

[5] Kattah JC, Talkad AV, Wang DZ, Hsieh YH, Newman-Toker DE (2009). HINTS to diagnose stroke in the acute vestibular syndrome: three-step bedside oculomotor examination more sensitive than early MRI diffusion-weighted imaging. Stroke.

[6] Buttner-Ennever JA (2008). Mapping the oculomotor system. Prog Brain Res.

[7] Brandt T, Dieterich M (1987). Pathological eye-head coordination in roll: tonic ocular tilt reaction in mesencephalic and medullary lesions. Brain.

[8] Dieterich M, Brandt T (1993). Ocular torsion and perceived vertical in oculomotor, trochlear and abducens nerve palsies. Brain.

[9] Dieterich M, Brandt T (1992). Wallenberg’s syndrome: lateropulsion, cyclorotation and subjective visual vertical in thirty-six patients. Ann Neurol.

[10] Dieterich M, Brandt T (1993). Ocular torsion and tilt of subjective visual vertical are sensitive brainstem signs. Ann Neurol.

[11] Halmagyi GM, Brandt T, Dieterich M, Curthoys IS, Stark RJ, Hoyt WF (1990). Tonic contraversive ocular tilt reaction due to unilateral meso-diencephalic lesion. Neurology.

[12] Brandt T, Dieterich M (1991). Different types of skew deviation. J Neurol Neurosurg Psychiatry.

[13] Ko MW, Dalmau J, Galetta SL (2008). Neuro-ophthalmologic manifestations of paraneoplastic syndromes. J Neuroophthalmol.

[14] Shallo-Hoffmann J, Schwarze H, Simonsz HJ, Muhlendyck H (1990). A reexamination of end-point and rebound nystagmus in normals. Invest Ophthalmol Vis Sci.

[15] Marti S, Straumann D, Glasauer S (2005). The origin of downbeat nystagmus: an asymmetry in the distribution of on-directions of vertical gaze-velocity purkinje cells. Ann N Y Acad Sci.

[16] Salsano E, Umeh C, Rufa A, Pareyson D, Zee DS (2012). Vertical supranuclear gaze palsy in Niemann–Pick type C disease. Neurol Sci.

[17] Halmagyi GM, Curthoys IS (1988). A clinical sign of canal paresis. Arch Neurol.

[18] Halmagyi GM, Curthoys IS (2000). A clinical sign of canal paresis. Arch Neurol.

[19] Bartl K, Lehnen N, Kohlbecher S, Schneider E (2009). Head impulse testing using video-oculography. Ann N Y Acad Sci.

[20] Gauthier GM, Vercher JL (1990). Visual vestibular interaction: vestibulo-ocular reflex suppression with head-fixed target fixation. Exp Brain Res.

[21] Chen AL, Riley DE, King SA, Joshi AC, Serra A, Liao K, Cohen ML, Otero-Millan J, Martinez-Conde S, Strupp M, Leigh RJ (2010). The disturbance of gaze in progressive supranuclear palsy: implications for pathogenesis. Front Neurol.

[22] Vanier MT (2013). Niemann–Pick diseases. Handb Clin Neurol.

[23] Garver WS, Francis GA, Jelinek D, Shepherd G, Flynn J, Castro G, Walsh VC, Coppock DL, Pettit KM, Heidenreich RA, Meaney FJ (2007). The National Niemann–Pick C1 disease database: report of clinical features and health problems. Am J Med Genet A.

[24] King G, Sharom FJ (2012). Proteins that bind and move lipids: MsbA and NPC1. Crit Rev Biochem Mol Biol.

[25] Poirier S, Mayer G, Murphy SR, Garver WS, Chang TY, Schu P, Seidah NG (2013). The cytosolic adaptor AP-1A is essential for the trafficking and function of Niemann–Pick type C proteins. Traffic.

[26] Sevin M, Lesca G, Baumann N, Millat G, Lyon-Caen O, Vanier MT, Sedel F (2007). The adult form of Niemann–Pick disease type C. Brain.

[27] Abel LA, Walterfang M, Fietz M, Bowman EA, Velakoulis D (2009). Saccades in adult Niemann–Pick disease type C reflect frontal, brainstem, and biochemical deficits. Neurology.

[28] Solomon D, Winkelman AC, Zee DS, Gray L, Buttner-Ennever J (2005). Niemann–Pick type C disease in two affected sisters: ocular motor recordings and brain-stem neuropathology. Ann N Y Acad Sci.

[29] Walterfang M, Macfarlane MD, Looi JC, Abel L, Bowman E, Fahey MC, Desmond P, Velakoulis D (2012). Pontine-to-midbrain ratio indexes ocular-motor function and illness stage in adult Niemann–Pick disease type C. Eur J Neurol.

[30] Patterson MC, Mengel E, Wijburg FA, Muller A, Schwierin B, Drevon H, Vanier MT, Pineda M (2013). Disease and patient characteristics in NP-C patients: findings from an international disease registry. Orphanet J Rare Dis.

[31] Klunemann HH, Santosh PJ, Sedel F (2012). Treatable metabolic psychoses that go undetected: what Niemann–Pick type C can teach us. Int J Psychiatry Clin Pract.

[32] Patterson MC, Hendriksz CJ, Walterfang M, Sedel F, Vanier MT, Wijburg F (2012). Recommendations for the diagnosis and management of Niemann–Pick disease type C: an update. Mol Genet Metab.

[33] Patterson MC, Vecchio D, Prady H, Abel L, Wraith JE (2007). Miglustat for treatment of Niemann–Pick C disease: a randomised controlled study. Lancet Neurol.

[34] Pineda M, Wraith JE, Mengel E, Sedel F, Hwu WL, Rohrbach M, Bembi B, Walterfang M, Korenke GC, Marquardt T, Luzy C, Giorgino R, Patterson MC (2009). Miglustat in patients with Niemann–Pick disease Type C (NP-C): a multicenter observational retrospective cohort study. Mol Genet Metab.

[35] Wraith JE, Baumgartner MR, Bembi B, Covanis A, Levade T, Mengel E, Pineda M, Sedel F, Topcu M, Vanier MT, Widner H, Wijburg FA, Patterson MC (2009). Recommendations on the diagnosis and management of Niemann–Pick disease type C. Mol Genet Metab.

[36] Hollak CE (2012). An evidence-based review of the potential benefits of taliglucerase alfa in the treatment of patients with Gaucher disease. Core Evid.

[37] Benko W, Ries M, Wiggs EA, Brady RO, Schiffmann R, FitzGibbon EJ (2011). The saccadic and neurological deficits in type 3 Gaucher disease. PLoS One.

[38] Desnick RJ (2004). Enzyme replacement and enhancement therapies for lysosomal diseases. J Inherit Metab Dis.

[39] Schiffmann R, FitzGibbon EJ, Harris C, DeVile C, Davies EH, Abel L, van Schaik IN, Benko W, Timmons M, Ries M, Vellodi A (2008). Randomized, controlled trial of miglustat in Gaucher’s disease type 3. Ann Neurol.

[40] Cox TM (2010). Eliglustat tartrate, an orally active glucocerebroside synthase inhibitor for the potential treatment of Gaucher disease and other lysosomal storage diseases. Curr Opin Investig Drugs.

[41] Doneda D, Netto CB, Moulin CC, Schwartz IV (2013). Effects of imiglucerase on the growth and metabolism of Gaucher disease type I patients: a systematic review. Nutr Metab (Lond).

[42] Neudorfer O, Pastores GM, Zeng BJ, Gianutsos J, Zaroff CM, Kolodny EH (2005). Late-onset Tay-Sachs disease: phenotypic characterization and genotypic correlations in 21 affected patients. Genet Med.

[43] Grosso S, Farnetani MA, Berardi R, Margollicci M, Galluzzi P, Vivarelli R, Morgese G, Ballestri P (2003). GM2 gangliosidosis variant B1 neuroradiological findings. J Neurol.

[44] Walterfang M, Bonnot O, Mocellin R, Velakoulis D (2013). The neuropsychiatry of inborn errors of metabolism. J Inherit Metab Dis.

[45] Osher E, Fattal-Valevski A, Sagie L, Urshanski N, Amir-Levi Y, Katzburg S, Peleg L, Lerman-Sagie T, Zimran A, Elstein D, Navon R, Stern N, Valevski A (2011). Pyrimethamine increases beta-hexosaminidase A activity in patients with Late Onset Tay Sachs. Mol Genet Metab.

[46] Salman MS, Ikeda KM (2013). The syndrome of infantile-onset saccade initiation delay. Can J Neurol Sci.

[47] Harris CM, Shawkat F, Russell-Eggitt I, Wilson J, Taylor D (1996). Intermittent horizontal saccade failure (‘ocular motor apraxia’) in children. Br J Ophthalmol.

[48] Le Ber I, Moreira MC, Rivaud-Pechoux S, Chamayou C, Ochsner F, Kuntzer T, Tardieu M, Said G, Habert MO, Demarquay G, Tannier C, Beis JM, Brice A, Koenig M, Durr A (2003). Cerebellar ataxia with oculomotor apraxia type 1: clinical and genetic studies. Brain.

[49] Le Ber I, Bouslam N, Rivaud-Pechoux S, Guimaraes J, Benomar A, Chamayou C, Goizet C, Moreira MC, Klur S, Yahyaoui M, Agid Y, Koenig M, Stevanin G, Brice A, Durr A (2004). Frequency and phenotypic spectrum of ataxia with oculomotor apraxia 2: a clinical and genetic study in 18 patients. Brain.

[50] Panouilleres M, Frismand S, Sillan O, Urquizar C, Vighetto A, Pelisson D, Tilikete C (2013). Saccades and eye-head coordination in ataxia with oculomotor apraxia type 2. Cerebellum.

[51] Durr A (2010). Autosomal dominant cerebellar ataxias: polyglutamine expansions and beyond. Lancet Neurol.

[52] Klockgether T, Paulson H (2011). Milestones in ataxia. Mov Disord.

[53] Gispert S, Twells R, Orozco G, Brice A, Weber J, Heredero L, Scheufler K, Riley B, Allotey R, Nothers C (1993). Chromosomal assignment of the second locus for autosomal dominant cerebellar ataxia (SCA2) to chromosome 12q23-24.1. Nat Genet.

[54] Geiner S, Horn AK, Wadia NH, Sakai H, Buttner-Ennever JA (2008). The neuroanatomical basis of slow saccades in spinocerebellar ataxia type 2 (Wadia-subtype). Prog Brain Res.

[55] Kim JS, Kim JS, Youn J, Seo DW, Jeong Y, Kang JH, Park JH, Cho JW (2013). Ocular motor characteristics of different subtypes of spinocerebellar ataxia: distinguishing features. Mov Disord.

[56] Rub U, Brunt ER, Seidel K, Gierga K, Mooy CM, Kettner M, Van Broeckhoven C, Bechmann I, La Spada AR, Schols L, den Dunnen W, de Vos RA, Deller T (2008). Spinocerebellar ataxia type 7 (SCA7): widespread brain damage in an adult-onset patient with progressive visual impairments in comparison with an adult-onset patient without visual impairments. Neuropathol Appl Neurobiol.

[57] Strupp M, Teufel J, Habs M, Feuerecker R, Muth C, van de Warrenburg BP, Klopstock T, Feil K (2013). Effects of acetyl-dl-leucine in patients with cerebellar ataxia: a case series. J Neurol.

[58] Strupp M, Thurtell MJ, Shaikh AG, Brandt T, Zee DS, Leigh RJ (2011). Pharmacotherapy of vestibular and ocular motor disorders, including nystagmus. J Neurol.

[59] Aure K, de Ogier BH, Laforet P, Jardel C, Eymard B, Lombes A (2007). Chronic progressive ophthalmoplegia with large-scale mtDNA rearrangement: can we predict progression?. Brain.

[60] DeBrosse S, Ubogu EE, Yaniglos S, Hassan MO, Leigh RJ (2009). Dynamic properties of eye movements in mitochondrial chronic progressive external ophthalmoplegia. Eye (Lond).

[61] Ritchie AE, Griffiths PG, Chinnery PF, Davidson AW (2010). Eye movement recordings to investigate a supranuclear component in chronic progressive external ophthalmoplegia: a cross-sectional study. Br J Ophthalmol.

[62] Moraes CT, DiMauro S, Zeviani M, Lombes A, Shanske S, Miranda AF, Nakase H, Bonilla E, Werneck LC, Servidei S (1989). Mitochondrial DNA deletions in progressive external ophthalmoplegia and Kearns-Sayre syndrome. N Engl J Med.

[63] Compain C, Sacre K, Puechal X, Klein I, Vital-Durand D, Houeto JL, De BT, Raoult D, Papo T (2013). Central nervous system involvement in Whipple disease: clinical study of 18 patients and long-term follow-up. Medicine (Baltimore).

[64] Schwartz MA, Selhorst JB, Ochs AL, Beck RW, Campbell WW, Harris JK, Waters B, Velasco ME (1986). Oculomasticatory myorhythmia: a unique movement disorder occurring in Whipple’s disease. Ann Neurol.

[65] Louis ED (2003). Whipple disease. Curr Neurol Neurosci Rep.

[66] Wagner JN, Glaser M, Brandt T, Strupp M (2008). Downbeat nystagmus: aetiology and comorbidity in 117 patients. J Neurol Neurosurg Psychiatry.

[67] Strupp M, Kremmyda O, Brandt T (2013). Pharmacotherapy of vestibular disorders and nystagmus. Semin Neurol.

[68] Strupp M, Schuler O, Krafczyk S, Jahn K, Schautzer F, Buttner U, Brandt T (2003). Treatment of downbeat nystagmus with 3,4-diaminopyridine: a placebo-controlled study. Neurology.

[69] Kalla R, Deutschlander A, Hufner K, Stephan T, Jahn K, Glasauer S, Brandt T, Strupp M (2006). Detection of floccular hypometabolism in downbeat nystagmus by fMRI. Neurology.

[70] Claassen J, Spiegel R, Kalla R, Faldon M, Kennard C, Danchaivijitr C, Bardins S, Rettinger N, Schneider E, Brandt T, Jahn K, Teufel J, Strupp M, Bronstein A (2013). A randomised double-blind, cross-over trial of 4-aminopyridine for downbeat nystagmus—effects on slowphase eye velocity, postural stability, locomotion and symptoms. J Neurol Neurosurg Psychiatry.

[71] Kalla R, Glasauer S, Buttner U, Brandt T, Strupp M (2007). 4-aminopyridine restores vertical and horizontal neural integrator function in downbeat nystagmus. Brain.

[72] Etzion Y, Grossman Y (2001). Highly 4-aminopyridine sensitive delayed rectifier current modulates the excitability of guinea pig cerebellar Purkinje cells. Exp Brain Res.

[73] Alvina K, Khodakhah K (2010). The therapeutic mode of action of 4-aminopyridine in cerebellar ataxia. J Neurosci.

[74] Dieterich M, Straube A, Brandt T, Paulus W, Buttner U (1991). The effects of baclofen and cholinergic drugs on upbeat and downbeat nystagmus. J Neurol Neurosurg Psychiatry.

[75] Glasauer S, Kalla R, Buttner U, Strupp M, Brandt T (2005). 4-aminopyridine restores visual ocular motor function in upbeat nystagmus. J Neurol Neurosurg Psychiatry.

